# Novel risk scores for survival and intracranial failure in patients treated with radiosurgery alone to melanoma brain metastases

**DOI:** 10.1186/s13014-015-0553-y

**Published:** 2015-12-01

**Authors:** Imran H. Chowdhury, Eric Ojerholm, Matthew T. McMillan, Denise Miller, James D. Kolker, Goldie Kurtz, Jay F. Dorsey, Suneel N. Nagda, Geoffrey A. Geiger, Steven Brem, Donald M. O’Rourke, Eric L. Zager, Tara Gangadhar, Lynn Schuchter, John Y. K. Lee, Michelle Alonso-Basanta

**Affiliations:** Department of Radiation Oncology, University of Pennsylvania, 3400 Civic Center Boulevard - TRC 2 West, Philadelphia, 19104 PA USA; Department of Neurosurgery, University of Pennsylvania, 3400 Spruce Street - 3 Silverstein, Philadelphia, 19104 PA USA; Department of Surgery, University of Pennsylvania, 3400 Spruce Street, 4 Silverstein, Philadelphia, 19104 PA USA; Department of Medicine, Division of Hematology/Oncology, University of Pennsylvania, 3400 Civic Center Boulevard, Philadelphia, 19104 PA USA

**Keywords:** Stereotactic radiosurgery, Melanoma, Brain metastases, Risk score

## Abstract

**Purpose:**

Stereotactic radiosurgery (SRS) alone is an increasingly common treatment strategy for brain metastases. However, existing prognostic tools for overall survival (OS) were developed using cohorts of patients treated predominantly with approaches other than SRS alone. Therefore, we devised novel risk scores for OS and distant brain failure (DF) for melanoma brain metastases (MBM) treated with SRS alone.

**Methods and materials:**

We retrospectively reviewed 86 patients treated with SRS alone for MBM from 2009-2014. OS and DF were estimated using the Kaplan-Meier method. Cox proportional hazards modeling identified clinical risk factors. Risk scores were created based on weighted regression coefficients. OS scores range from 0-10 (0 representing best OS), and DF risk scores range from 0-5 (0 representing lowest risk of DF). Predictive power was evaluated using c-index statistics. Bootstrapping with 200 resamples tested model stability.

**Results:**

The median OS was 8.1 months from SRS, and 54 (70.1 %) patients had DF at a median of 3.3 months. Risk scores for OS were predicated on performance status, extracranial disease (ED) status, number of lesions, and gender. Median OS for the low-risk group (0-3 points) was not reached. For the moderate-risk (4-6 points) and high-risk (6.5-10) groups, median OS was 7.6 months and 2.4 months, respectively (*p* < .0001). Scores for DF were predicated on performance status, ED status, and number of lesions. Median time to DF for the low-risk group (0 points) was not reached. For the moderate-risk (1-2 points) and high-risk (3-5 points) groups, time to DF was 4.8 and 2.0 months, respectively (*p* < .0001). The novel scores were more predictive (c-index = 0.72) than melanoma-specific graded prognostic assessment or RTOG recursive partitioning analysis tools (c-index = 0.66 and 0.57, respectively).

**Conclusions:**

We devised novel risk scores for MBM treated with SRS alone. These scores have implications for prognosis and treatment strategy selection (SRS versus whole-brain radiotherapy).

## Introduction

Melanoma brain metastases (MBM) are a common type of secondary intracranial neoplasm and will develop in nearly half of patients with advanced cutaneous melanoma [1–3]. The rate of MBM is likely to rise given the increasing incidence of melanoma and advances in systemic disease control with targeted therapies [4, 5]. The overall survival (OS) of these patients is generally poor, and many suffer a neurologic death [2, 6, 7].

Radiotherapy treatment options for MBM include whole-brain radiation therapy (WBRT) and stereotactic radiosurgery (SRS) [8]. WBRT irradiates both the known metastases and potential microscopic disease — maximizing intracranial control but at the cost of neurotoxicity [7, 9–14]. Focal SRS targets only the visible disease and spares the remaining brain; however, there is an increased risk of new distant brain metastases with SRS alone, which can independently impact cognition [15–19]. While the optimal strategy remains controversial, SRS alone is an increasingly common treatment approach, particularly for patients with a limited volume of metastatic disease [20].

In order to tailor treatment to individual patients, several important prognostic tools have been created for patients with brain metastases. In 1997, Gaspar et al. [21] analyzed 1200 patients from three Radiation Therapy Oncology Group (RTOG) trials. Using recursive partitioning analysis (RPA), three classes were devised which stratified patients based on survival. The RPA classes were further improved by Sperduto et al. in 2008 with the creation of the graded prognostic assessment (GPA) [22]. Neither tool, however, was specific for primary disease histology. Recognizing the prognostic variances of different tumor types, a set of disease-specific GPAs were then devised [23]. These included a melanoma-GPA, which identified performance status and number of MBM as prognostic for survival. One limitation of the melanoma-GPA is the widely heterogeneous treatment approaches in the development cohort. Patients were managed with WBRT alone, SRS alone, WBRT plus SRS, surgery followed by WBRT, surgery followed by SRS, or a combination of all three modalities. Importantly, the majority of patients were treated with a strategy other than SRS alone. Even with these existing tools, the ability to predict survival in SRS patients remains poor [24].

Therefore, this study analyzed MBM patients treated solely with SRS and sought to create risk scores for survival that could improve upon the existing melanoma-GPA. Secondary aims included identifying predictors of distant brain failure, potentially identifying patients who may benefit from WBRT.

## Methods and materials

### Data collection

With approval of the institutional review board, this study retrospectively identified 86 consecutive patients with intact MBM treated with SRS alone from 2009 to 2014 at the University of Pennsylvania. Patient, disease, and treatment characteristics were retrieved from electronic medical records and GammaPlan software treatment records. Primary cutaneous melanoma diagnosis was recorded at the first date of histologically confirmed melanoma. Mutation status was classified as BRAF wild-type (WT) or BRAF mutation, including V600E, K601E, or V600K. Brain metastasis diagnosis was defined as the date of first metastatic disease on brain magnetic resonance imaging (MRI) or computed tomography (CT).

Extracranial disease (ED) status was categorized as active, stable, or absent based on CT scans of the chest, abdomen and pelvis or positron emission tomography/computed tomography (PET/CT) within two months of SRS. Active ED indicated patients with new or increasing burden of metastatic melanoma to solid organs outside the brain, including patients with newly diagnosed MBM with co-existing extracranial metastases. Stable ED denoted patients with previously treated extracranial metastases with either a partial response or stable size/metabolic activity. Absent ED indicated patients with no history of extracranial metastases or previously treated extracranial disease with complete radiographic response. RPA class and melanoma-GPA score were assigned according to Gaspar et al. [21] and Sperduto et al., [23] respectively.

GammaPlan software was used to retrospectively record MBM tumor volumes and SRS treatment volumes. Tumor volumes of individual lesions were obtained from the SRS planning T1-weighted, contrast-enhanced MRI. To avoid inter-planner variability, a single investigator (I.C.) with attending supervision (M.A.B) — both blinded to OS and distant failure (DF) data — retrospectively contoured each lesion. Treatment volumes of individual metastases were defined as the volume of brain tissue receiving at least the prescribed marginal dose for each MBM. Total tumor and treatment volumes were calculated by summing all respective individual volumes. Systemic therapy was classified as peri-SRS if administered at the time of SRS or completed within two months of SRS. Systemic therapy was alternatively designated as post-SRS if administered during the interval between SRS and DF, or if it was the first therapy given after SRS in patients without DF. Dates of death were determined from the Social Security Death Index, hospice records, and local newspaper obituaries.

### Stereotactic radiosurgery

Radiosurgery was performed using the Model 4-C or Perfexion GK (Elekta Inc., Stockholm, Sweden) with GammaPlan software. A Leksell stereotactic headframe was applied with local anesthesia, and high-resolution brain MR images were taken at 1-mm slices with gadolinium contrast. Additional new MBM discovered on the planning images were targeted with SRS in the same session. Per institutional standards, post-SRS follow-up brain MRI was obtained approximately every 2 months for 1 year and then every 3 months afterward.

### Statistical analysis

All 86 patients were included in the OS analysis, with living patients or those lost to follow-up censored at the date of last clinical encounter. DF was analyzed in 77 (89.5 %) patients with follow-up imaging. The remaining nine (10.5 %) patients died or were lost to follow-up prior to first post-treatment intracranial imaging. DF was defined as leptomeningeal disease or new parenchymal MBM at sites other than previous treatment. Patients free of any failure were censored at the date of last imaging showing intracranial control.

The median follow-up time was computed using the inverse Kaplan-Meier method [25], while OS and time to DF were estimated using the Kaplan-Meier method. Variables with a *p-*value ≤0.10 on univariate analysis were considered for multivariable analysis. For clinical utility, continuous variables were categorized using previously described techniques [26]. Prior to modeling, correlations between variables were checked for multicollinearity. Missing data points (i.e., BRAF mutation status, N = 15) were addressed via multivariable imputation.

Two different multivariable models were developed to identify predictors of OS and DF. The first used all variables that demonstrated significance during univariate Cox proportional hazards modeling, while the second used a backwards, stepwise elimination procedure (exit criteria: p > 0.05) to identify the most parsimonious model. Bootstrapping with 200 resamples was used to test model stability and control for over-optimism.

Next, significant factors relative to each outcome of interest were used to derive risk scores for OS and DF. Point values were assigned to each risk factor based on weighted regression coefficients from the Cox proportional hazards model. Reference categories for each variable were assigned zero points. Scores were tabulated for each patient based on the presence of weighted risk factors, and patients were then sub-classified into clinically useful risk groups. Patients were assigned to one of three risk groups for OS based on similarities in survival patterns. This process was repeated for DF risk; group assignments for DF risk were made independently of the patient’s designation for OS risk.

Model discrimination was evaluated using the Harrell’s concordance index (c-index) [27] for both OS and DF. A c-index value of 0.5 indicates no predictive capacity, while 1.0 indicates perfect discrimination. Harrell’s c-indices were also calculated for the melanoma-GPA and RPA for OS. These results were compared to the c-index derived from our SRS-specific MBM OS risk scores.

Descriptive statistics were performed for all variables. P-values ≤0.05 were considered statistically significant. Statistical computations were performed utilizing IBM SPSS, version 22 (IBM Corp., Armonk, NY) and SAS 9.3 (SAS Institute Inc., Cary, NC).

## Results

### Patient characteristics

Patient and disease characteristics are presented in Table 1. The cohort was predominantly male (60.5 %) with a Karnofsky performance status (KPS) ≥80 (91.8 %) and a median age of 56 years at SRS. Mutation status was obtained in 71 (82.5 %) patients and not available in 15 (17.4 %) patients. ED status was determined by CT in 48 (55.8 %) patients and PET/CT in 38 (44.1 %) patients within a median of 2.64 weeks prior to SRS. Patients underwent SRS to a median of two (interquartile range [IQR] 1-4) metastases. Fifty (58.1 %) patients received systemic therapy peri-SRS, while the remaining 36 (41.9 %) did not receive any at the time of SRS. According to previously devised prognostic tools for survival [21, 28], the majority of patients were classified as RPA class II (89.5 %), while melanoma-GPA categories were more evenly distributed.

**Table 1:** Patient characteristics and treatment data

Characteristics	No. of patients:	Median	Range
	*n* = 86 (%)		
Age at GK, years		56	(24–90)
Gender			
Male	52 (60.5)		
Female	34 (39.5)		
Extracranial Metastases			
Active	64 (74.4)		
Stable	13 (15.1)		
None	9 (10.5)		
Chemotherapy at SRS			
Yes	50 (58.1)		
Temozolmide	13 (15.1)		
Ipilimumab	11 (12.8)		
BRAF-inhibitor	9 (10.5)		
None	36 (41.9)		
No. of lesions			
1	27 (31.4)		
2	25 (29.1)		
3	9 (10.5)		
4	5 (5.8)		
5	7 (8.1)		
6	4 (4.7)		
≥7	9 (10.5)		
KPS			
<80	7 (8.1)		
≥80	79 (91.8)		
RPA Class			
I	6 (6.9)		
II	77 (89.5)		
III	3 (3.5)		
Melanoma-GPA points			
3.5-4.0	22 (25.6)		
2.5-3.0	35 (40.7)		
1.5-2.0	26 (30.2)		
0-1.0	3 (3.5)		
Mutation Status			
BRAF WT	31 (36.0)		
BRAF V600E, K601E, V600K	37 (43.0)		
c-kit	2 (2.3)		
NRAS	1 (1.2)		
N/A	15 (17.4)		
Total tumor volume, cc		1.5	0.01–34.4
<3 cc	61 (70.9)		
≥3 cc	25 (29.1)		
Marginal GK dose, Gy		21	12–22
Total treatment volume, cc		3.6	0.1–45.2
<7	65 (75.6)		
≥7	21 (24.4)		

Abbreviations: *GK* Gamma Knife, *KPS* Karnofsky performance status, *RPA* recursive partitioning analysis, *Melanoma-GPA* graded prognostic assessment for melanoma, *BRAF* B-Raf proto-oncogene, *BRAF WT* B-Raf proto-oncogene wild-type, *c-KIT* c-kit proto-oncogene, *NRAS* neuroblastoma RAS viral (v-ras) oncogene homolog, *Gy* Gray, *cc* cubic centimeter

### Overall survival

With a median follow-up of 37.4 (IQR 13.8–47.8) months, median OS for the cohort was 8.1 (IQR 4.0-19.2) months from SRS. On univariate analysis, factors associated with worse OS included: KPS ≤80, presence of any ED (absent vs. stable/active), presence of active ED (absent/stable vs. active), 2-4 lesions, >4 lesions, and not receiving post-SRS systemic therapy. Presence of a BRAF mutation was not associated with worse OS compared to BRAF WT. On multivariable analysis, KPS ≤80 (HR 8.1, *P* < .0001), presence of any ED (absent vs. stable/active: HR 5.4, *P* = .05), presence of 2-4 lesions (HR 2.6, *P* = .04) and >4 lesions (HR 3.2, *P* = .002), remained significantly associated with worse OS (Table 2). Although male gender was not significant on univariate analysis, stepwise regression of all variables identified gender as being significant (HR 1.8, *P* = .03), even when subjected to bootstrapping. Therefore, male gender was included as a component of the OS risk score. RPA class and melanoma-GPA scores were not included in the multivariable analysis in order to avoid collinearity.

**Table 2:** Univariate and multivariable Cox proportional hazards analysis for overall survival

Prognostic factor	Univariate analysis		Multivariable analysis	
	Hazard ratio (95 % CI)	*P* value	Hazard ratio (95 % CI)	*P* value
Age (continuous)	1.02 (1.00-1.03)	0.064		
Age (>70 or ≤ 70)^a^	1.52 (0.89-2.58)	0.123		
Gender (female or male)^b^	1.3 (0.81-2.22)	0.241	1.76 (1.04-2.97)	0.034
KPS (>80 or ≤ 80)	5.16 (2.55-10.45)	<.0001	8.09 (3.79-17.28)	0.001
Extracranial disease (absent vs. stable/active)	5.27 (1.64-16.94)	0.005	5.37 (1.65-17.48)	0.002
Extracranial disease (absent/stable vs. active)	2.31 (1.24-4.29)	0.008		
No. of lesions				
1	1.00			
2-4	2.01 (1.09-3.71)	0.025	2.61 (1.37-4.96)	0.004
>4	2.98 (1.47-6.03)	0.002	3.24 (1.56-6.72)	0.002
BRAF (WT vs. mutation)	0.99 (0.57-1.70)	0.960		
Metastases volume (<1 cc vs. ≥1 cc)	1.70 (1.02-2.82)	0.041		
Treatment volume (<4 cc vs. ≥4 cc)	1.66 (1.02-2.70)	0.042		
Peri-SRS systemic therapy (no vs. yes)	1.49 (0.92-2.43)	0.109		
Post-SRS systemic therapy (no vs. yes)	0.41 (0.20-0.85)	0.016		

Abbreviations: *BRAF* B-Raf proto-oncogene, *CI* 95 % confidence interval, *KPS* Karnosfky performance status, *peri-SRS* at the time of stereotactic radiosurgery, *post-SRS* after stereotactic radiosurgery, *WT* wild-type, ^a^cut-off at the upper quartile of age; ^b^stepwise regression of all variables identified gender as being significant, therefore, it was included on multivariable analysis

**Fig. 1:**
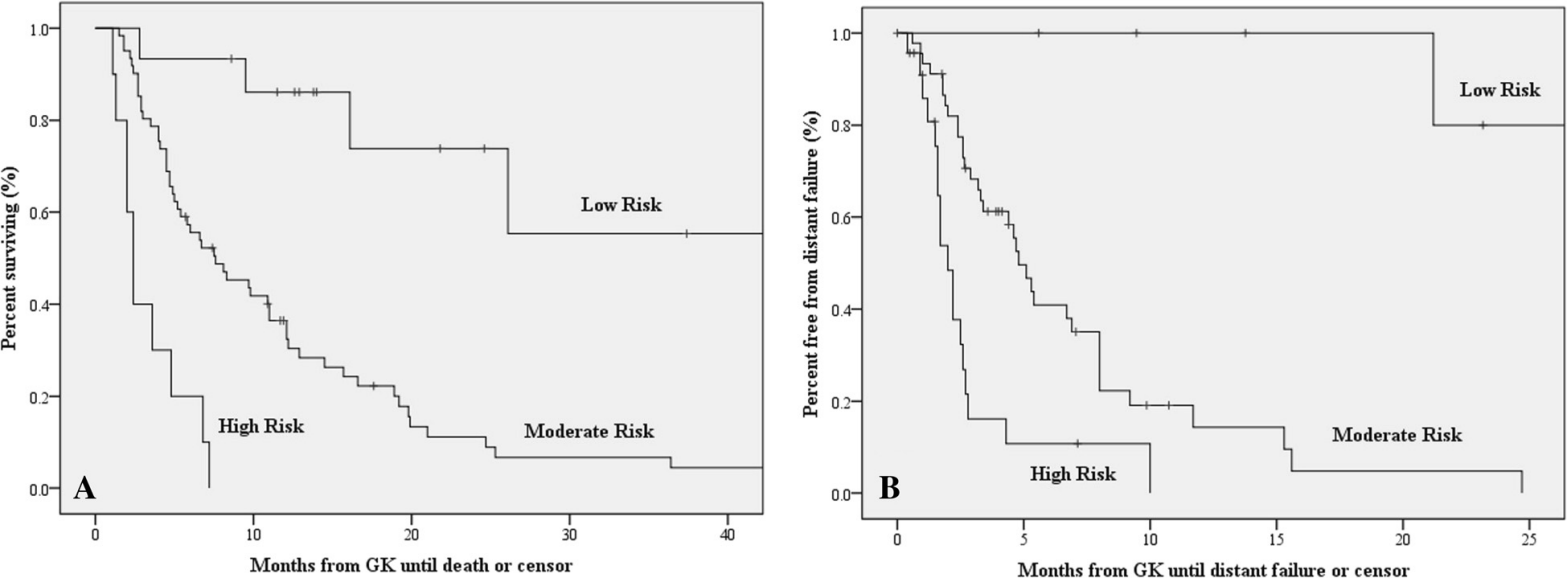
**a** Kaplan-Meier curves for overall survival for low-risk (0-3 points), moderate-risk (4-6 points) and high-risk (6.5-10 points) survival groups. **b** Kaplan-Meier curves for distant failure for low-risk (0 points), moderate-risk (1-2 points) and high-risk (3-5 points) distant failure groups

Values for the scoring system were determined from the weighted proportions of hazard ratios (Table 3). The most heavily weighted risk factor was KPS ≤80, followed by presence of any ED, number of lesions (2-4 or >4), and gender. Total point values could range from 0-10, with 0 representing best OS and 10 representing worst OS. After tabulating individual scores, patients were classified into 3 risk groups (low, moderate, and high) based on similar survival patterns. OS differed significantly between the groups (*P* < .0001 using the log-rank test for all pairwise comparisons between groups). Median OS estimates for risk groups are represented in the bottom portion of Table 3. A visual representation of OS between the different risk groups is shown in Fig. 1, panel a. The novel risk scores had a higher Harrell’s C index (c-index = 0.72) than the melanoma-GPA (c-index = 0.66) and the RPA (c-index = 0.57).

**Table 3:** Point scoring system of predictive factors and median overall survival by risk zones

Prognostic factor		Multivariable analysis hazard ratio (95 % CI)	Point score
KPS			
>80		1.00	0
≤80		8.09 (3.79-17.28)	4
Extracranial disease			
absent		1.00	0
active/stable		5.37 (1.65-17.48)	3
No. of lesions			
1		1.00	0
2-4		2.61 (1.37-4.96)	1.5
>4		3.24 (1.56-6.72)	2
Gender			
Female		1.00	0
Male		1.76 (1.04-2.97)	1
Risk zone	Point ranges	No. of patients (%)	Median OS, mo (95 % CI)
Low	0-3	15 (17.4)	not reached
Moderate	4-6	61 (71.7)	7.60 (3.73-11.47)
High	6.5-10	10 (11.6)	2.40 (1.79-3.01)

Abbreviations: *CI* 95 % confidence interval, *KPS* Karnofsky performance status, *OS* overall survival

### Distant failure

Of the 77 patients who had follow-up imaging, 54 (70.1 %) patients had DF at a median of 3.3 (IQR 1.8-7.1) months from SRS. Median follow-up for these patients was 13.8 (IQR 5.6-40.7) months. Factors associated with DF on univariate analysis included: KPS ≤80, presence of any ED (absent vs. stable/active), presence of active ED (absent/stable vs. active), 2-3 lesions, and >3 lesions. On multivariable analysis, KPS ≤80 (HR 4.4, *P* = .004), presence of any ED (HR 2.6, *P* = .008), 2-3 lesions (HR 2.3, *P* = .032), and >3 lesions (HR 5.9, *P* < .0001) remained significantly associated with DF (Table 4). Stepwise regression confirmed these findings.

**Table 4:** Univariate and multivariable Cox proportional hazards analysis for distant failure

	Univariate analysis		Multivariable analysis	
Prognostic factor	Hazard ratio (95 % CI)	*P* value	Hazard ratio (95 % CI)	*P* value
Age (continuous)	1.00 (0.98-1.02)	0.872		
Age (>70 or ≤ 70)^a^	1.10 (0.59-2.07)	0.760		
Gender (female or male)	0.89 (0.52-1.53)	0.675		
KPS (>80 or ≤ 80)	3.20 (1.21-8.48)	0.019	4.37 (1.58-12.06)	0.004
Extracranial disease (absent vs. stable/active)	2.63 (1.04-6.68)	0.041	2.60 (1.29-5.27)	0.008
Extracranial disease (absent/stable vs. active)	2.58 (1.33-5.01)	0.005		
No. of lesions				
1	1.00			
2-3	3.13 (1.48-6.61)	0.003	2.30 (1.08-4.91)	0.032
>3	5.93 (2.65-13.26)	<.0001	5.27 (2.40-11.57)	<.0001
BRAF (WT vs. mutation)	0.89 (0.49-1.63)	0.713		
Metastases volume (<1 cc vs. ≥1 cc)	1.47 (0.85-2.54)	0.171		
Treatment volume (<4 cc vs. ≥4 cc)	1.70 (0.99-2.92)	0.054		
Peri-SRS systemic therapy (no vs. yes)	1.48 (0.87-2.54)	0.150		
Post-SRS systemic therapy (no vs. yes)	0.74 (0.29-1.89)	0.534		

Abbreviations: *BRAF* B-Raf proto-oncogene, *CI* 95 % confidence interval, *KPS* Karnosfky performance status, *peri-SRS* at the time of stereotactic radiosurgery, *post-SRS* after stereotactic radiosurgery, *WT* wild-type; ^a^cut-off at the upper quartile of age

A methodological approach similar to the one used to create the OS risk score was applied to calculate patients’ risk of DF. DF risk scores ranged from 0-5, with 0 representing the lowest risk of DF and 5 representing the greatest risk for DF (Table 5). Risk groups for DF (low, moderate, and high) had significantly different patterns of recurrence (*P* < .0001 using the log-rank test for all pairwise comparisons between groups). The median time to DF for the risk groups are represented in the bottom portion of Table 5. Similar to the OS risk model, the risk groups derived from the DF model demonstrated a high predictive capacity (c-index = 0.72). A visual representation of DF between the different risk groups is shown in Fig. 1, panel b.

**Table 5:** Point scoring system of predictive factors and median time to distant failure by risk zones

Prognostic factor		Multivariable analysis hazard ratio (95 % CI)	Point score
KPS			
>80		1.00	0
≤80		4.37 (1.58-12.06)	2
Extracranial disease			
absent		1.00	0
active/stable		2.60 (1.29-5.27)	1
No. of lesions			
1		1.00	0
2-3		2.30 (1.08-4.91)	1
>3		5.27 (2.40-11.57)	2
Risk zone	Point ranges	No. of patients (%)	Median time to DF, mo (95 % CI)
Low	0	8 (9.3)	not reached
Moderate	1-2	46 (53.4)	4.80 (3.87-5.73)
High	3-5	23 (26.7)	2.00 (1.50-2.50)

Abbreviations: *CI* 95 % confidence interval, *KPS* Karnofsky performance status, *DF* distant failure

### Systemic therapy & adverse events

Systemic therapy analysis included the 77 patients with follow-up imaging, of which 67 (87 %) patients received systemic therapy, while 10 (13 %) patients did not. When stratifying systemic therapy among the OS risk groups, 14 (93 %) patients in the low-risk group received post-SRS systemic therapy, compared to 50 (89 %) and 3 (50 %) in the moderate- and high-risk groups, respectively. It should be noted that 5 patients from the moderate OS risk group and 4 patients from the high OS risk group were lost to follow-up imaging and were not included in systemic therapy analysis. Several therapies were predominately administered: ipilimumab (40 %) and/or temozolomide (33 %) in the low-risk group; temozolomide (23 %), vemurafinib (16 %) and/or ipilimumab (14 %) in the moderate-risk group; and no therapy (50 %) in the high-risk group. Univariate analysis demonstrated significant association between OS and receipt of post-SRS systemic therapy (*P* = .016), but was not significant after adjusting for other factors. There was no univariate association of DF and post-SRS systemic therapy (*P* = .066). Nine (11.7 %) patients with intracranial disease only (no stable or active ED), had a median OS of 21.8 months, 7 of which received post-SRS systemic therapy (3 ipilimumab, 3 temozolomide, and 1 vemurafenib).

Among the risk groups for DF, all the patients in the low-risk group received post-SRS systemic therapy, with 50 % receiving temozolomide and 38 % receiving ipilimumab. In the moderate-risk group, an equal percentage (20 %) received ipilimumab, temozolomide, or vemurafenib. An equal percentage (26 %) of high-risk patients received either temozolomide or no therapy, while 13 % of patients received ipilimumab. Among patients with follow-up, 7 (9.1 %) had symptomatic radiation necrosis and 9 (11.7 %) patients had hemorrhagic metastases.

## Discussion

In this study, we devised novel risk scores for overall survival and distant brain failure for patients with MBM treated with SRS alone. In the survival risk score, performance status, presence of any ED (active or stable), number of lesions, and gender were clinical predictors of survival in descending significance. Three risk zones were defined (Table 3), partitioning patients into groups with significantly different expected survival. The new survival score had a higher Harrell’s C index than either of the RPA classes or the melanoma-GPA (c-index = 0.72, versus 0.57 and 0.66, respectively). Those existing tools were developed in patient cohorts treated predominantly with strategies other than SRS alone; however, patients selected for an SRS-only approach tend to have unique characteristics such as high performance status and low-to-moderate intracranial metastatic burden. These cases may reflect an inherently different disease biology compared to patients recommended for treatment with WBRT or multimodality combinations, as seen in the melanoma-GPA cohort.

In our distant brain failure score, performance status, presence of any ED (active or stable), and number of lesions were factors associated with DF on multivariable analysis. Three risk zones were defined (Table 5), partitioning patients into groups with significantly different expected intracranial failure rates. Prior investigations of MBM support the inclusion of ED status [29, 30] and number of metastases [30–32] as predictors of DF. Recently, Huttenlocher et al. also developed a tool for estimating DF in SRS-treated MBM [33]. Their final model included ED status and number of lesions, but their patient cohort was limited to cases with 1-3 metastases. Recent evidence suggests that SRS may be appropriate for greater than three metastases with low overall tumor burden [29, 34–36], and our tool extends the ability of clinicians to estimate DF for this population.

Despite existing prognostic tools, physicians are not able to accurately judge survival for many patients treated with SRS for brain metastases [24]. The current study may improve this prognostic ability. Survival estimates are important anticipatory information for patients and may assist in weighing the relative risks and benefits of treatment. Furthermore, existing tools (i.e. melanoma GPA and RPA) are only prognostic for OS. However, assessing the risk of DF may be important when considering whether to use SRS or WRBT [37]. In our novel DF model, patients in the low-risk group may benefit from SRS alone, which controls the index lesions while minimizing the volume of irradiated brain. This may prevent the neurocognitive side effects associated with WBRT [38–40]. On the other hand, patients at high risk of new metastases may benefit more from micrometastatic disease control with WBRT. Several studies have reported that intracranial progression causes more severe neurocognitive deficits than exposure to WBRT [15–19]. Therefore WBRT may best preserve cognitive functioning in the specific group of patients with high risk of DF. Patients in the moderate risk-zone may require a more individualized treatment plan based on the larger clinical picture. Since the use of SRS alone for brain metastases is increasing [20], our OS and DF models will help clinicians accurately assess prognosis in a new era of treatment, while identifying a subset of patients that may benefit more with WBRT than SRS alone.

This study has several limitations, including the biases inherent to a retrospective investigation. Additionally, although both models were internally validated using bootstrapping methodology, the risk scores were developed using a single institution cohort; therefore, a subsequent external validation is necessary to confirm the generalizability of the findings. Furthermore, systemic therapy is also rapidly evolving for melanoma, and it is possible that newer agents (e.g., immune checkpoint and BRAF inhibitors) could become part of standard treatment in the future. Several phase II trials have shown the potential activity of these inhibitors for MBM [41, 42]. Recent retrospective analyses suggest a benefit of combining SRS with Ipilimumab; for example, Kiess et al. demonstrated that concurrent combination of Ipilimumab at the time of SRS was associated with improved local control and survival [43, 44]. These preliminary results are being tested in phase III trials of Ipilimumab (clinicaltrials.gov NCT01703507 and NCT01950195) and Debrafenib (clinicaltrials.gov NCT01721603) in conjunction with SRS. Additionally, systemic therapy varied widely in our patients. Therefore, the model’s accuracy may be different in cohorts with an alternative distribution of systemic therapy. However, systemic therapy was not significantly associated with OS or DF in this study, and our patient cohort represents contemporary treatment trends. Finally, neurocognitive data was not collected and thus hindered incorporation of neurologic deaths into our analysis. Many of these limitations will be addressed by an open phase III randomized trial assessing MBM treated with SRS with or without WBRT [45, 46].

## Conclusion

In conclusion, this study developed novel risk scores for survival and distant brain failure in patients with MBM treated with SRS alone. The survival score demonstrated a higher predictive capacity than existing tools such as RPA class or melanoma-GPA, and the distant failure score identifies a subset of patients who may benefit from WBRT more than SRS.

## Abbreviations

MBM: Melanoma brain metastases
OS: Overall survival
DF: Distant failure
WBRT: Whole-brain radiation therapy
SRS: Stereotactic radiosurgery
RTOG: Radiation therapy oncology group
RPA: Recursive partitioning analysis
GPA: Graded prognostic assessment
WT: Wild-type
MRI: Magnetic resonance imaging
CT: Computed tomography
ED: Extracranial disease
PET/CT: Positron emission tomography/computed tomography
KPS: Karnofsky performance status
Gy: Gray
CC: Cubic centimeter
IQR: Interquartile range
HR: Hazards ratio

## References

[1] Barnholtz-Sloan JS, Sloan AE, Davis FG, Vigneau FD, Lai P, Sawaya RE (2004). Incidence proportions of brain metastases in patients diagnosed (1973 to 2001) in the Metropolitan Detroit Cancer Surveillance System. J Clin Oncol.

[2] Davies MA, Liu P, McIntyre S, Kim KB, Papadopoulos N, Hwu WJ (2011). Prognostic factors for survival in melanoma patients with brain metastases. Cancer.

[3] Nayak L, Lee EQ, Wen PY (2012). Epidemiology of brain metastases. Curr Oncol Rep.

[4] Curti BD (2014). Rapid evolution of combination therapy in melanoma. N Engl J Med.

[5] Siegel R, Ma J, Zou Z, Jemal A (2014). Cancer statistics, 2014. CA Cancer J Clin.

[6] Sampson JH, Carter JH, Friedman AH, Seigler HF (1998). Demographics, prognosis, and therapy in 702 patients with brain metastases from malignant melanoma. J Neurosurg.

[7] Chang EL, Selek U, Hassenbusch SJ, 3rd, Maor MH, Allen PK, Mahajan A et al. Outcome variation among “radioresistant” brain metastases treated with stereotactic radiosurgery. Neurosurgery. 2005;56(5):936-45; discussion -45. 15854241

[8] Gorantla V, Kirkwood JM, Tawbi HA (2013). Melanoma brain metastases: an unmet challenge in the era of active therapy. Curr Oncol Rep.

[9] Andrews DW, Scott CB, Sperduto PW, Flanders AE, Gaspar LE, Schell MC (2004). Whole brain radiation therapy with or without stereotactic radiosurgery boost for patients with one to three brain metastases: phase III results of the RTOG 9508 randomised trial. Lancet.

[10] Aoyama H, Shirato H, Tago M, Nakagawa K, Toyoda T, Hatano K (2006). Stereotactic radiosurgery plus whole-brain radiation therapy vs stereotactic radiosurgery alone for treatment of brain metastases: a randomized controlled trial. JAMA.

[11] Brown PD, Brown CA, Pollock BE, Gorman DA, Foote RL (2008). Stereotactic radiosurgery for patients with “radioresistant” brain metastases. Neurosurgery.

[12] Kocher M, Soffietti R, Abacioglu U, Villa S, Fauchon F, Baumert BG (2011). Adjuvant whole-brain radiotherapy versus observation after radiosurgery or surgical resection of one to three cerebral metastases: results of the EORTC 22952-26001 study. J Clin Oncol.

[13] Kondziolka D, Patel A, Lunsford LD, Kassam A, Flickinger JC (1999). Stereotactic radiosurgery plus whole brain radiotherapy versus radiotherapy alone for patients with multiple brain metastases. Int J Radiat Oncol Biol Phys.

[14] Patchell RA, Tibbs PA, Walsh JW, Dempsey RJ, Maruyama Y, Kryscio RJ (1990). A randomized trial of surgery in the treatment of single metastases to the brain. N Engl J Med.

[15] Aoyama H, Tago M, Kato N, Toyoda T, Kenjyo M, Hirota S (2007). Neurocognitive function of patients with brain metastasis who received either whole brain radiotherapy plus stereotactic radiosurgery or radiosurgery alone. Int J Radiat Oncol Biol Phys.

[16] Li J, Bentzen SM, Renschler M, Mehta MP (2007). Regression after whole-brain radiation therapy for brain metastases correlates with survival and improved neurocognitive function. J Clin Oncol.

[17] Meyers CA, Smith JA, Bezjak A, Mehta MP, Liebmann J, Illidge T (2004). Neurocognitive function and progression in patients with brain metastases treated with whole-brain radiation and motexafin gadolinium: results of a randomized phase III trial. J Clin Oncol.

[18] Regine WF, Huhn JL, Patchell RA, St Clair WH, Strottmann J, Meigooni A (2002). Risk of symptomatic brain tumor recurrence and neurologic deficit after radiosurgery alone in patients with newly diagnosed brain metastases: results and implications. Int J Radiat Oncol Biol Phys.

[19] Regine WF, Scott C, Murray K, Curran W (2001). Neurocognitive outcome in brain metastases patients treated with accelerated-fractionation vs. accelerated-hyperfractionated radiotherapy: an analysis from Radiation Therapy Oncology Group Study 91-04. Int J Radiat Oncol Biol Phys.

[20] Choosing Wisely® An Initiative of the ABIM Foundation: American Society for Radiation Oncology, 10 Things Physicians and Patients Should Question Web site ABIM Foundation. 2013. http://www.choosingwisely.org/societies/american-society-for-radiation-oncology/.

[21] Gaspar L, Scott C, Rotman M, Asbell S, Phillips T, Wasserman T (1997). Recursive partitioning analysis (RPA) of prognostic factors in three Radiation Therapy Oncology Group (RTOG) brain metastases trials. Int J Radiat Oncol Biol Phys.

[22] Sperduto PW, Berkey B, Gaspar LE, Mehta M, Curran W (2008). A new prognostic index and comparison to three other indices for patients with brain metastases: an analysis of 1960 patients in the RTOG database. Int J Radiat Oncol Biol Phys.

[23] Sperduto PW, Kased N, Roberge D, Xu Z, Shanley R, Luo X (2012). Summary report on the graded prognostic assessment: an accurate and facile diagnosis-specific tool to estimate survival for patients with brain metastases. J Clin Oncol.

[24] Kondziolka D, Parry PV, Lunsford LD, Kano H, Flickinger JC, Rakfal S (2014). The accuracy of predicting survival in individual patients with cancer. J Neurosurg.

[25] Schemper M, Smith TL (1996). A note on quantifying follow-up in studies of failure time. Control Clin Trials.

[26] Mazumdar M, Glassman JR (2000). Categorizing a prognostic variable: review of methods, code for easy implementation and applications to decision-making about cancer treatments. Stat Med.

[27] Harrell FE, Lee KL, Mark DB (1996). Multivariable prognostic models: issues in developing models, evaluating assumptions and adequacy, and measuring and reducing errors. Stat Med.

[28] Gaspar LE, Scott C, Murray K, Curran W (2000). Validation of the RTOG recursive partitioning analysis (RPA) classification for brain metastases. Int J Radiat Oncol Biol Phys.

[29] Liew DN, Kano H, Kondziolka D, Mathieu D, Niranjan A, Flickinger JC (2011). Outcome predictors of Gamma Knife surgery for melanoma brain metastases. Clinical article. J Neurosurg.

[30] Yu C, Chen JC, Apuzzo ML, O'Day S, Giannotta SL, Weber JS (2002). Metastatic melanoma to the brain: prognostic factors after gamma knife radiosurgery. Int J Radiat Oncol Biol Phys.

[31] Dyer MA, Arvold ND, Chen YH, Pinnell NE, Mitin T, Lee EQ (2014). The role of whole brain radiation therapy in the management of melanoma brain metastases. Radiat Oncol.

[32] Selek U, Chang EL, Hassenbusch SJ, Shiu AS, Lang FF, Allen P (2004). Stereotactic radiosurgical treatment in 103 patients for 153 cerebral melanoma metastases. Int J Radiat Oncol Biol Phys.

[33] Huttenlocher S, Sehmisch L, Schild SE, Blank O, Hornung D, Rades D (2014). Identifying melanoma patients with 1-3 brain metastases who may benefit from whole-brain irradiation in addition to radiosurgery. Anticancer Res.

[34] Nabors LB, Portnow J, Ammirati M, Brem H, Brown P, Butowski N (2014). Central nervous system cancers, version 2.2014. Featured updates to the NCCN Guidelines. J Natl Compr Canc Netw.

[35] Yamamoto M, Serizawa T, Shuto T, Akabane A, Higuchi Y, Kawagishi J (2014). Stereotactic radiosurgery for patients with multiple brain metastases (JLGK0901): a multi-institutional prospective observational study. Lancet Oncol.

[36] Ojerholm E, Lee JY, Kolker J, Lustig R, Dorsey JF, Alonso-Basanta M (2014). Gamma Knife radiosurgery to four or more brain metastases in patients without prior intracranial radiation or surgery. Cancer Med.

[37] Ojerholm E, Alonso-Basanta M, Simone CB (2014). Stereotactic radiosurgery alone for small cell lung cancer: a neurocognitive benefit?. Radiat Oncol.

[38] Chang EL, Wefel JS, Hess KR, Allen PK, Lang FF, Kornguth DG (2009). Neurocognition in patients with brain metastases treated with radiosurgery or radiosurgery plus whole-brain irradiation: a randomised controlled trial. Lancet Oncol.

[39] Gondi V, Paulus R, Bruner DW, Meyers CA, Gore EM, Wolfson A (2013). Decline in tested and self-reported cognitive functioning after prophylactic cranial irradiation for lung cancer: pooled secondary analysis of Radiation Therapy Oncology Group randomized trials 0212 and 0214. Int J Radiat Oncol Biol Phys.

[40] Soffietti R, Kocher M, Abacioglu UM, Villa S, Fauchon F, Baumert BG (2013). A European Organisation for Research and Treatment of Cancer phase III trial of adjuvant whole-brain radiotherapy versus observation in patients with one to three brain metastases from solid tumors after surgical resection or radiosurgery: quality-of-life results. J Clin Oncol.

[41] Hauschild A, Grob J-J, Demidov LV, Jouary T, Gutzmer R, Millward M et al. Dabrafenib in BRAF-mutated metastatic melanoma: a multicentre, open-label, phase 3 randomised controlled trial. The Lancet.380(9839):358-65. doi:10.1016/S0140-6736(12)60868-X. 10.1016/S0140-6736(12)60868-X 22735384

[42] Margolin K, Ernstoff MS, Hamid O, Lawrence D, McDermott D, Puzanov I (2012). Ipilimumab in patients with melanoma and brain metastases: an open-label, phase 2 trial. Lancet Oncol.

[43] Kiess AP, Wolchok JD, Barker CA, Postow MA, Tabar V, Huse JT et al. Stereotactic Radiosurgery for Melanoma Brain Metastases in Patients Receiving Ipilimumab: Safety Profile and Efficacy of Combined Treatment. Int J Radiat Oncol Biol Phys. 2015;92(2):368-75. doi:10.1016/j.ijrobp.2015.01.004. 10.1016/j.ijrobp.2015.01.004 PMC4955924 25754629

[44] Knisely JP, Yu JB, Flanigan J, Sznol M, Kluger HM, Chiang VL (2012). Radiosurgery for melanoma brain metastases in the ipilimumab era and the possibility of longer survival. J Neurosurg.

[45] Fogarty G, Morton RL, Vardy J, Nowak AK, Mandel C, Forder PM (2011). Whole brain radiotherapy after local treatment of brain metastases in melanoma patients--a randomised phase III trial. BMC Cancer.

[46] Fogarty GB, Hong A, Jacobsen KD, Reisse CH, Shivalingam B, Burmeister B (2014). Accrual to a randomised trial of adjuvant whole brain radiotherapy for treatment of melanoma brain metastases is feasible. BMC Res Notes.

